# How Does Conscientiousness Relate to Employee Creativity? An Exploratory Study of Frontline Technical Workers †

**DOI:** 10.3390/bs15020201

**Published:** 2025-02-13

**Authors:** Sen Xu, Jiajia Cheng

**Affiliations:** School of Economics and Management, Nanjing Tech University, Nanjing 211816, China; xusen_nj@njtech.edu.cn

**Keywords:** employee creativity, conscientiousness, perceived time pressure, trust in one’s leader

## Abstract

Building on the interactionist perspective of organizational creativity, we investigated the relationship between conscientiousness and employee creativity. We surveyed 260 frontline technical workers and their supervisors at three Chinese manufacturing enterprises. The results showed a positive relationship between conscientiousness and employee creativity. We also found a three-way interaction effect between conscientiousness, perceived time pressure, and trust in one’s leader, which impacts employee creativity. We ended by discussing both the theoretical and managerial implications.

## 1. Introduction

Employee creativity is essential for organizational effectiveness and development (Anderson et al., 2014; Zhou & Shalley, 2003). Organizational scholars have long taken an interactionist perspective (Woodman et al., 1993; Zhou & Hoever, 2014) to examine how some explicitly creativity-relevant personality traits, for example, openness to experience, could interact with certain work environments to enhance employee creativity (Anderson et al., 2014; Zhou & Hoever, 2014; Zhou & Shalley, 2003). However, not every organization can fill all of its positions with individuals possessing creativity-relevant personalities. Thus, we might need alternative mindsets to exploit the creative potentials of those who are not typically seen to be creative and positioned in creativity-demanding places.

Conscientious employees, for instance, are known to be accountable, planned, persistent, and achievement-oriented. Though conscientious employees have been consistently recognized as good performers at work (Barrick & Mount, 1991), they are rarely assumed to develop high levels of employee creativity (Anderson et al., 2014; Zhou & Shalley, 2003). Only a handful of researchers have looked at the relationship between conscientiousness and employee creativity (George & Zhou, 2001; Miron et al., 2004; Raja & Johns, 2010; Wang et al., 2012; Woods et al., 2018). Their findings are far from conclusive. Also, almost none of these empirical studies have investigated the creativity of conscientious employees in less creativity-relevant positions. Aware of the insufficient research on employees with less traditional creativity-relevant features, we direct our attention to the frontline employees whose creative potentials are not expected but actually essential for organizational effectiveness and development.

In this regard, we examine whether conscientiousness could affect employee creativity in those in less creativity-relevant positions. We propose that conscientiousness could encourage these frontline employees to take on creative tasks as a part of their role. Also, we investigate two contextual factors that may moderate the relationship between conscientiousness and employee creativity: the perceived time pressure and trust in one’s leader. Frontline employees may be subject to extra stress and complexity associated with the requirement for creative work (Elsbach & Hargadon, 2006). As sufficient time is important to encourage employee creativity (Amabile et al., 1996), the perceived time pressure could highly influence these employees’ willingness and involvement in creative work. Likewise, employee creativity may involve certain risk taking. Trust in one’s leader could free conscientious employees from anxiety when they choose to try novel approaches in the pursuit of excellence. Overall, our study investigates a theoretical framework that includes the positive relationship between the conscientiousness and creativity of frontline employees, the moderating roles of the perceived time pressure and trust in one’s leader, and the interactive effects of the two moderators.

Our potential contributions to the creativity literature are twofold. First, we respond to recent suggestions of investigating the relationship between personality traits and individual creativity (Feist et al., 2017). We attempt to answer calls for studies on how conscientiousness, recognized as one of the less creativity-relevant personality traits, influences employee creativity in certain work and social contexts (Anderson et al., 2014; Zhou & Hoever, 2014). Second, our study enriches the interactionist perspective of organizational creativity (Woodman et al., 1993; Zhou & Hoever, 2014) by substantiating evidence that contextual conditions could encourage conscientious employees to work creatively. We cater to a growing interest in the ambivalent role of the perceived time pressure in improving employee creativity (e.g., Amabile et al., 1996; Ohly et al., 2006) and the effect of trust in one’s leader on employee creativity (George & Zhou, 2007; Gong et al., 2013; Harris et al., 2014; Javed et al., 2018; Zhang & Zhou, 2014). In particular, our findings unfold the complex nature of the person–context interactive effects on employee creativity (Zhou & Hoever, 2014).

## 2. Theoretical Development and Hypotheses

### 2.1. Conscientiousness and Employee Creativity

Psychologists have drawn close attention to the Big Five personality trait model (extraversion, neuroticism, agreeableness, conscientiousness, and openness to experience) (Costa & McCrae, 1992; Costa et al., 1991) to explain individual differences in creativity (Feist et al., 2017). Feist (1998) conducted the first meta-analytic review on Big Five personality traits and individual creativity. He found that creative individuals are likely to be more open to experiences, less conscientious, somewhat extraverted, less agreeable, and have more neuroticism. Later, Feist (2017) updated his analysis and suggested that almost all five dimensions of the Big Five personality traits could somewhat enhance individual creativity in different environments.

Conscientiousness is known as one of the Big Five personality traits most theoretically relevant to productivity and achievement (Costa & McCrae, 1992). There were quite mixed results regarding to what extent conscientiousness influences individual creativity (for reviews, see Batey & Furnham, 2006; Feist, 1998, 2017; Furst & Lubart, 2017; Reiter-Palmon et al., 2009). For instance, Feist (1998) suggested that conscientiousness is one of the most stable Big Five personality traits relevant to individual creativity. Artists were described as less conscientious, whereas scientists were more conscientious. Ivcevic (2007) found that conscientious college students have high levels of creativity. Yet Batey et al. (2010) suggested a negative association between the conscientiousness and ideational behavior (an indicator for individual creativity) of college students in the United Kingdom and the United States. A recent cross-cultural study indicates a nonsignificant relationship between conscientiousness and the creative performance of undergraduate students in both China and the United States (Deng et al., 2016).

Similarly, the relationship between conscientiousness and employee creativity is inconclusive. Prior research suggests that conscientiousness is conceptually relevant to employee creativity (Zhou & Shalley, 2003). Based on their high performance, conscientious employees can transform their experiences into strong creative self-efficacy, which in turn enhances their creativity (Liu et al., 2016). But this relationship is not consistent (Shalley et al., 2004); conscientiousness can either promote or hinder employee creativity in different contexts (Anderson et al., 2014). It is advisable that the interactionist perspective can be used as “a guiding principle” to examine how specific personal characteristics like conscientiousness interact with contextual factors to influence employees’ creativity (Zhou & Shalley, 2003). In other words, the effect of conscientiousness on employee creativity may vary in work and social contexts. For instance, George and Zhou (2001) found that highly conscientious employees exhibit low individual creativity when they work with unsupportive coworkers and with supervisors who monitor staff closely. Woods et al. (2018) identified that conscientious British financial institution employees exhibited lower innovative work behaviors when these employees had a longer organizational tenure. Wang et al. (2012) found that highly conscientious employees could exhibit high individual creativity when they worked in organizations with outcome- and innovation-oriented cultures. In direct contrast to the Wang et al. (2012) study, Miron et al. (2004) did not find that conscientious employees displayed a high individual innovative performance when they worked in organizational cultures dominated by either innovation or attention to detail, or were outcome-oriented. However, Raja and Johns (2010) did not find that the job scope (that is, the extent to which a job is rich, challenging, and complex) affects the relationship between conscientious employees and their individual creativity.

Given the contextual awareness of the relationship between conscientiousness and employee creativity, we wonder whether creativity-relevant personality characteristics might overshadow conscientiousness in existing research since a majority of studies have focused on college students or personnel engaged in highly creativity-demanding positions. To raise the visibility of the relationship between conscientiousness and employee creativity, we choose to focus on the employee creativity of frontline employees. Though the creative potentials of these employees have been less attended to in previous creativity literature, these workers usually undertake medium-complexity work that may provide them with appropriately flexible task boundaries for performing their jobs. Also, their firms and supervisors may expect, encourage, or demand them to make some creative adaptations to their workflow for better work efficiency and effectiveness. Though the employee creativity might be a high-standard requirement for frontline employees, conscientiousness will encourage them to accept creative assignments as a part of their goals at work so as to satisfy their basic psychological need for competence.

Conscientiousness includes two aspects: striving for achievement (hard-working and achievement-oriented) and being dependable (careful, dutiful, and organized) (Barrick & Mount, 1991; Haller & Courvoisier, 2010). We believe that conscientiousness could be either beneficial or undermining to the creativity of frontline employees for two reasons. First, conscientious employees are usually more enthusiastic about achievement and excellence (Barrick & Mount, 1991). Thus, highly conscientious frontline employees have the stronger internal desire to improve their effectiveness than less conscientious frontline employees do. They will actively aim to meet the expectations of their organizations and supervisors who may request their employees to either significantly change or slightly improve existing techniques or procedures. This improvement is what we conceptualize as creativity within the role and scope of the employees.

In addition, conscientious employees, given their well-organized and dutiful natures (Barrick & Mount, 1991), would cautiously organize their workload to balance routine and novelty in performance. Also, they may take a dynamic view of routines by adding some innovative work into their fixed schedules (Ohly et al., 2006). They are more likely to take a dynamic view of routines, thereby including routines as an innate aspect of employee creativity (Sonenshein, 2016). Such careful and orderly resource allocation enables them to fulfill their autonomy and personal relatedness in order to demonstrate their creative work capacity.

By putting all these arguments together, we propose our first hypothesis:

**Hypothesis 1.**  *There is a positive relationship between conscientiousness and the creativity of frontline employees.*

### 2.2. The Moderating Effect of Perceived Time Pressure

The interactionist perspective of organizational creativity suggests that employee creativity is a consequence of interactions between individual and contextual factors (Woodman et al., 1993; Zhou & Hoever, 2014). In this study, we focus on one work factor (time pressure) and one social factor (trust in one’s leader) that may influence conscientious employees to develop employee creativity.

The perceived time pressure refers to “the perception that there is a scarcity of time available to complete a task, or set of tasks, relative to the demands of the task(s) at hand” (Maruping et al., 2015, p. 1315). We used the perceived time pressure to indicate to what extent conscientious employees could control for their own work time to achieve employee creativity. Specifically, frontline employees choose the best strategies to complete their creative work under the conditions of time pressure they perceive by the deadlines. According to activation theory (Gardner, 1990), the perceived time pressure is a challenge stressor that can optimally stimulate conscientious employees to consider whether or not and the extent to which they may fulfill their task creatively. Specifically, the perceived time pressure provides highly relevant work context for occupations where employee creativity is not a compulsory requirement of a job (e.g., frontline technical workers).

Based on prior studies of time pressure on employee creativity from the perspective of activation theory (Baer & Oldham, 2006; Binnewies & Wörnlein, 2011), we propose that a moderate level of perceived time pressure could positively influence conscientious employees’ willingness to use new work procedures or approaches to enhance their work effectiveness and efficiency (Trougakos et al., 2014), which consequently achieves their personal goals such as work engagement (Kühnel et al., 2012) and work–life balance (Unger et al., 2014).

When time pressure is at a low level, conscientious frontline employees may not be motivated to work creatively for more effectiveness and efficiency because existing routine and procedures can achieve good task performance. They may use their remaining resources (e.g., effort and concentration) to achieve a more routinized task performance rather than creative outputs. As time pressure moves from low to moderate levels, conscientious employees may start to experience considerable time pressure and think about how to complete their assignments more creatively (Byron et al., 2010). As time pressure moves from moderate to high levels, conscientious employees have to cope with an enormous workload and exhaust their energy. Their preference for responsibility, order, and routine might push conscientious employees to comply with existing procedures as a safer way of completing work on time rather than taking risky creative approaches. In sum, we hypothesize that time pressure might have a nonlinear moderating effect on how conscientious employees perform creatively:

**Hypothesis 2.**  *Perceived time pressure moderates the relationship between conscientiousness and employee creativity in a curvilinear format, such that this relationship is more positive for frontline employees when they perceive moderate levels of time pressure than either low or high levels of time pressure.*

### 2.3. The Moderating Effect of Trust in One’s Leader

Trust in one’s leader refers to employees’ willingness to be vulnerable towards their supervisors, based on employees’ positive expectations of their supervisors’ behavior (Mayer et al., 1995). It is known to be one of the positive contextual factors combined with other stimuli (e.g., pay for performance, mood, and leadership) to activate employee creativity (e.g., George & Zhou, 2007; Gong et al., 2013; Harris et al., 2014; Javed et al., 2018; Zhang & Zhou, 2014). Trust in one’s leader serves as a high standard of social support beyond the role of typical organizational support for employee creativity that could influence the relationship between conscientiousness and employee creativity. Specifically, conscientious employees are particularly reliant on their direct leaders who can provide positive feedback that implicitly recognizes and even approves their investigation on creative outputs during daily work. Such recognition and approval is essential because it helps those conscientious employees facilitate uncertainty and risk taking when they work creatively instead of achieving routinized task performance. Especially, those conscientious frontline employees generally receive organizational support for better routinized task performance rather than creative outputs. Thus, trust in one’s leader, particularly regarding direct supervisors, can enable the conscientious technical workers’ willingness to work creatively.

Conscientious frontline employees are more likely to develop employee creativity when they have high levels of trust in their leader. They seem more likely to take creativity as a personal work goal because of high trust; thus, these employees are more likely to take supervisors’ perspectives into account. When they are asked to work creatively, those conscientious employees with high levels of trust would considerably demonstrate their creative capabilities because they do not want to disappoint their trusted supervisors. Conscientious frontline employees could have less concern about the potential risks of their creative attempts if they believe that their trusted supervisors have a variety of resources to support their creative effort (Mayer et al., 1995). With increasing levels of trust in their leaders, such conscientious employees are likely to explore either small adaptations or brand-new procedures because their trusted supervisors could authorize them to have more control of their work (Zhang & Zhou, 2014).

By contrast, conscientious frontline employees are less likely to exhibit employee creativity when they have low levels of trust in their leader. They may not take creative attempts seriously as a personal work goal. Without strong personal connections, these conscientious employees may have a reduced interest in, or make a reduced effort towards, adaptation or change and just follow their workplace routines. They may even abandon any form of creative change at work, even though these changes could potentially improve work effectiveness and efficiency. Also, conscientious employees are more vulnerable to doubting their creative competence because they wonder if their less trusted supervisors could provide the necessary emotional support to withstand difficulties (Harris et al., 2014). Such feelings of “indifference” could lead to conscientious employees’ dissatisfaction with their work environments and thus decrease their willingness to work creatively. Therefore, we propose our third hypothesis:

**Hypothesis 3.**  *Trust in one’s leader moderates the relationship between conscientiousness and employee creativity, such that this relationship is more positive for frontline employees with high levels of trust in their leader than for those with low levels of such trust.*

### 2.4. Interactive Efforts of Perceived Time Pressure and Trust in One’s Leader

The interactionist perspective of organizational creativity (Woodman et al., 1993; Zhou & Hoever, 2014) suggests that individual and situational contexts may have a complex interactive effect on employee creativity. Based on our previous discussion, we suggest that the curvilinear moderating effect of the perceived time pressure upon the relationship between conscientiousness and employee creativity would be stronger when those conscientious employees have high levels of trust in their leaders.

As we discussed before, conscientious employees with high levels of trust in their leaders are more open to risk taking and more keen on creative approaches for greater achievement. A moderate level of perceived time pressure could induce these conscientious employees with high levels of trust in their leaders to propose novel ideas for maximal productivity. On the contrary, when those conscientious frontline employees with high levels of trust in their leaders experience too low or too high a time pressure, their perceptions might not be beneficial to employee creativity. Perceiving low time pressure, those conscientious employees with high levels of trust in their leaders are less likely to bother their leaders for performance evaluation since new changes are not pressing to their current satisfactory work rhythm. Under high time pressure, the priority of even those conscientious employees with high levels of trust in their leaders’ experience might be the completion of their current work assignments in an orderly way. These employees are more likely to follow existing procedures for secure productivity since they feel confident that their trusted leaders will appreciate their decision making abilities when assessing their performance in time management. Therefore, we propose our fourth hypothesis:

**Hypothesis 4.**  *There is a three-way interaction between conscientiousness, time pressure, and trust in one’s leader on employee creativity, such that the curvilinear moderating effect of time pressure upon the relationship between conscientiousness and employee creativity will be stronger when those conscientious employees have high levels of trust in their leaders.*

## 3. Methods

### 3.1. Context

We conducted our study in an industrial park located in Hunan, China. We surveyed frontline technical workers in three manufacturing enterprises, based on their outstanding performance and large-scale operations. Also, these three enterprises encouraged or even demanded their workers to work creatively. Specifically, the three enterprises regularly held training workshops to enrich and update the knowledge of their workers. They also carried out quarterly competitions that encouraged the workers to generate creative ideas.

These enterprises are in different industries: plastics (Enterprise One), instruments and meters (Enterprise Two), and biological extraction technical equipment (Enterprise Three). Although all the workers in the three manufacturing enterprises undertook similar moderately complex assembly work assignments, they worked differently in terms of the products they were assembling. In Enterprise One, the workers collaborated with each other to assemble electronic toys in a sequential flow line; in Enterprise Two, each worker worked independently to assemble instruments and meters in machines; in Enterprise Three, each worker was assigned to arrange the work division. They manually operated machines to assemble parts of biological extraction technical equipment on three rotating shifts.

### 3.2. Sample and Procedures

We distributed separate questionnaires to 314 frontline technical workers and their 31 direct supervisors. Participants comprised approximately eighty percent, ninety percent, and seventy percent of the overall workers in each enterprise, respectively. One author administered the data collection. He asked the employees and their supervisors to fill in the surveys in separate time slots and locations during a working day. These participants completed the surveys onsite and sent them back to him directly.

We received responses from a total of 297 employees and 26 supervisors with a response rate of 94.6% for employees and 83.9% for supervisors. After deleting questionnaires with excessive missing data and those without matches between supervisors and employees, we received a total of 260 valid surveys from employees and 21 from supervisors for data analyses. The response rates were 82.8% (83.5%, 91.9%, and 77.6%, respectively) for employees and 67.7% (75%, 100%, and 61.1%, respectively) for supervisors. More than two-thirds of the employees were female (71.9%). Their average age and organizational tenure were 36 years and 3.18 years, respectively.

### 3.3. Measures

The surveys were translated into Mandarin Chinese. Two bilingual doctoral students majoring in management conducted the translation and back-translation process independently to ensure the accuracy of each item (Brislin, 1986).

Conscientiousness. We used three items adopted from Benet-Martinez and John’s (1998) Big Five Inventory. The employees rated themselves, ranging from one (does not apply to me at all) to six (applies to me perfectly). A sample item was “I do a thorough job.” Cronbach’s alpha for this scale was 0.85.

Perceived time pressure. We used Semmer et al.’s (1995) four-item Instrument for Stress-Oriented Task Analysis. We asked employees to describe their feelings about time pressure in the workplace, ranging from one (very rare) to six (very often). A sample item was “How often are you pressed for time?” Cronbach’s alpha for this scale was 0.85.

Trust in one’s leader. We adopted Mayer and Gavin’s (2005) five-item scale. The employees rated their trust in their direct supervisors, ranging from one (strongly disagree) to six (strongly agree). A sample item was “I would be willing to let my supervisor have complete control over my future in this company.” Cronbach’s alpha for this scale was 0.72.

Employee creativity. We used Farmer et al.’s (2003) four-item scale. We asked supervisors to rate the individual creativity of each employee, ranging from one (strongly disagree) to six (strongly agree). A sample item was “He/she tries new ideas or methods first.” Cronbach’s alpha for this scale was 0.97.

Control variables. First, we controlled for openness to experience, which has been identified as a key Big Five trait that enables employee creativity (George & Zhou, 2001). We used Benet-Martinez and John’s (1998) three-item scale. Employees estimated their openness to experience, from one (does not apply to me at all) to six (applies to me perfectly). A sample item was “He/she is original, comes up with new ideas.” Cronbach’s alpha for this scale was 0.83.

Second, we controlled for intrinsic motivation regarding its fundamental effect upon employee creativity. We used Gagné et al.’s (2015) scale. Employees rated three items from one (does not apply to me at all) to six (applies to me perfectly) by answering the question “Why do you make efforts in this job?” A sample item was “Because I have fun doing my job.” Cronbach’s alpha for this scale was 0.87.

Finally, we controlled for four demographic and organizational variables suggested by prior studies (e.g., Zhang & Zhou, 2014): (1) age (years), (2) gender (0 = female, 1 = male), (3) organizational tenure (years), and (4) organization. We created two dummy variables to control for the three organizations in the analyses.

## 4. Results

### 4.1. Confirmatory Factor Analyses

We conducted reliability and consistency tests on all variables by using SPSS 26.0 software. The composite reliability (CR) values for all the variables exceeded the recommended threshold of 0.7 (Hair et al., 2006), indicating satisfactory reliability. Additionally, all of the factor loadings in this study were above 0.5 (Hair et al., 2006). The average variance extracted (AVE) for all the constructs was higher than 0.5, except for the trust in one’s leader (AVE = 0.35). However, previous research has suggested that it is acceptable for a construct’s AVE to be below 0.5 if its CR exceeds 0.7 (Fornell & Larcker, 1981). So, all of the variables demonstrate good reliability and consistency.

We conducted a confirmatory factor analysis (CFA) to evaluate the distinctiveness of the primary constructs (conscientiousness, perceived time pressure, trust in one’s leader, and employee creativity), as well as two theoretical control variables (openness to experience and intrinsic motivation) with Mplus 7 (Muthén & Muthén, 1998–2015). The measurement model fit the data well: χ^2^ = 447.40, df = 194, comparative fit index (CFI) = 0.93, Tucker–Lewis index (TLI) = 0.92, root mean square error of approximation (RMSEA) = 0.07, standardized root mean square residual (SRMR) = 0.07, and all factor loadings were significant. We then compared this measurement model with several alternative models in which two or more factors were specified to correlate at unity. In each case, the fit decreased as indicated by the chi-square difference tests and model fit indices, which revealed that the six-factor model fit the data considerably better than any of the alternative models. Based on these results, we proceeded to analyze the study variables as distinct constructs.

### 4.2. Descriptive Statistics

Table 1 reports the mean, standard deviation, and correlation among the variables. The results show that the employee creativity was positively related to conscientiousness (r = 0.49, *p* < 0.01) and trust in one’s leader (r = 0.16, *p* < 0.05), but did not correlate to the perceived time pressure (r = −0.01, ns).

### 4.3. Hypothesis Testing

Since each supervisor rated the individual creativity of two or more employees, we first examined whether these supervisors rated independently. We ran an ANOVA test and found systematic differences in the supervisors’ ratings of employee creativity (F = 50.29, *p* < 0.001; ICC (1) = 0.80). We used multilevel modeling with Mplus 7 (Muthén & Muthén, 1998–2015) to conduct the path analysis. All of our variables were treated as Level 1 variables nested within supervisors at Level 2. All of the substantive variables were group mean-centered to ensure that we had correct interpretations of the main and the moderating effects (Enders & Tofighi, 2007; Preacher et al., 2016).

Table 2 reports the results of the multilevel regression analyses that were conducted to test the hypotheses. We introduced the control variables and examined the possibility of this linear relationship (Model 0). As shown in Table 2, the effect of conscientiousness on employee creativity was positive and significant (B = 0.16, *p* < 0.05). Thus, Hypothesis 1 was supported.

Hypothesis 2 predicted that the perceived time pressure has a nonlinear moderating effect on the positive relationship between conscientiousness and employee creativity, such that the relationship is more pronounced among those employees with medium levels of perceived time pressure than in either low or high levels of perceived time pressure. To test this hypothesis, we introduced the perceived time pressure (Model 1), perceived time pressure and its interaction with conscientiousness (Model 2), and perceived time pressure squared (Model 3), as well as perceived time pressure and its interaction with conscientiousness, and perceived time pressure squared and its interaction with conscientiousness (Model 4) into the regression equation. As shown in Table 2, the coefficient associated with the interaction term between conscientiousness and perceived time pressure squared was nonsignificant (B = −0.03, ns). Therefore, Hypothesis 2 was not supported.

Hypothesis 3 predicted that the trust in one’s leader moderates the positive relationship between conscientiousness and employee creativity, such that the relationship is more pronounced for those employees with high levels of trust in their leader than for those with low levels of trust in their leader. To test this hypothesis, we introduced trust in their leader (Model 5) as well as trust in their leader and its interactions with conscientiousness (Model 6) into the regression equation. As seen in Table 2, the coefficient associated with the interaction term for trust in their leader and conscientiousness was nonsignificant (B = −0.06, ns). Therefore, Hypothesis 3 was not supported.

Hypothesis 4 predicted that both the perceived time pressure and trust in their leader jointly moderate the relationship between conscientiousness and employee creativity. To test this interactive effect, we introduced a three-way interaction term (conscientiousness × perceived time pressure × trust in one’s leader; see Model 7 in Table 2) as well as a quadratic interaction term (conscientiousness × perceived time pressure squared × trust in their leader; see Model 8 in Table 2) into the regression equation. Table 2 shows that this three-way interaction term was negative and statistically significant (B = −0.08, *p* < 0.05).

**Figure 1:**
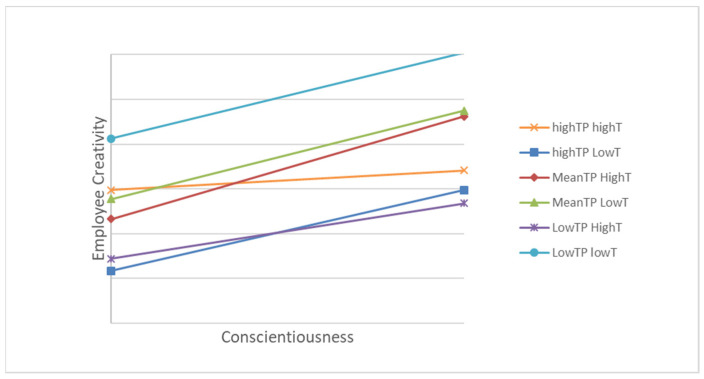
The three-way curvilinear interaction among conscientiousness, time pressure (TP), and trust in leader (T) on employee creativity.

By using Dawson’s (2014) approach, we drew up the interaction plot. As seen in Figure 1, a positive relationship appeared between conscientiousness and employee creativity in all conditions.

We conducted the simple slope analyses to specify the three-way quadratic interaction (Aiken & West, 1991; Dawson, 2014). The results show that the slope was positive and significant for those in the condition of both a low perceived time pressure and low trust in one’s leader (B = 0.19, SE = 0.06, *p* < 0.01), as well as those in the condition of both a moderate perceived time pressure and low trust in one’s leader (B = 0.20, SE = 0.08, *p* < 0.05), and those in the condition of both a high perceived time pressure and low trust in one’s leader (B = 0.18, SE = 0.06, *p* < 0.01). In the remaining conditions, the slopes were not significant. Therefore, Hypothesis 4 was partially supported.

## 5. Discussion

In this study, we examined the relationship between conscientious and employee creativity under certain working conditions. Our findings identified this main positive conscientiousness–employee creativity relationship and a three-way interaction relating to the perceived time pressure and trust in one’s leader.

### Theoretical Implications

Our study contributes to the literature. First, we found that conscientiousness could enhance employee creativity. The interactionist perspective of organizational creativity has taken personality traits as one important factor for employee creativity (Woodman et al., 1993; Zhou & Hoever, 2014). Researchers have suggested that highly conscientious employees tend to obey the existing techniques and procedures at work rather than work creatively (George & Zhou, 2001; Miron et al., 2004; Raja & Johns, 2010; Wang et al., 2012; Woods et al., 2018). By contrast, we investigated conditions that allow those conscientious employees to perform creatively and found a direct positive effect of conscientiousness on the employee creativity of frontline technical workers. Such empirical evidence shows that the relationship between conscientiousness and individual creativity may vary significantly in positions (Feist, 1998, 2017; Zhou & Shalley, 2003).

To our knowledge, this is the first study to find that conscientious frontline technical workers could manifest high levels of employee creativity. This underscores the necessity for scholars to unfold the intricate relationship about how one personality trait predicts employees’ creative performance (Anderson et al., 2014). We identify that conscientiousness could serve a crucial personal factor to facilitate the individual creativity of those employees who may not be traditionally assumed to have creativity-relevant personal characteristics (Anderson et al., 2014; Zhou & Shalley, 2003). Since the other four Big Five personality traits may also influence individual creativity (Feist, 2017), our finding encourages further research to investigate how broader contexts such as other occupations and industries provide appropriate work and social contexts to promote employee creativity (Zhou & Hoever, 2014).

Second, our results suggest that the perceived time pressure is one of the most ambiguous work contexts for employee creativity (Amabile et al., 1996). Its effect on the relationship between conscientiousness and employee creativity could be more complicated than what we assumed. This conforms to the views of Zhou and Hoever (2014), who state that, from the interactionist perspective of organizational creativity, the perceived time pressure might be categorized as a configurational factor, which is “hard or even impossible to classify as positive or negative…and involves certain factors that are not individually helpful or harmful but that specifically promote or hinder creativity in particular configurations with other factors” (p. 352). Zhou and Hoever (2014) suggested that these contexts for configurational factors could consist of an interaction or a combination of multiple contextual factors.

Finally, we clarify the relationship between trust in one’s leader and employee creativity. A few studies have identified that trust in one’s leader is one aspect of positive work conditions for employee creativity because employees feel safe about risk taking (George & Zhou, 2007; Harris et al., 2014; Javed et al., 2018; Zhang & Zhou, 2014). Our findings suggest that trust in one’s leader may not encourage conscientious technical workers to develop employee creativity despite the possibility that risky creative attempts may not undermine the leader–member relationship. It is possible that trust in one’s leader might not always work as a strong boundary condition to stimulate high employee creativity.

Thus, from the interactionist perspective of organizational creativity (Zhou & Hoever, 2014), there might exist a “diminishing gains” effect when one positive social context (e.g., trust in one’s leader) interacts with one positive personality trait (e.g., conscientiousness). Rather, trust in one’s leader should work with other work contextual factors to function effectively. In our study, though low trust in one’s leader may leave employees less friendly and offer less error-tolerant social contexts for creative ideas, we found that it could motivate conscientious technical workers to demonstrate employee creativity under certain work constraints like perceived time pressure. We expect additional research to explore the double-edged role of trust in one’s leader in employee creativity.

Our finding of three-way interaction effects also provides initial evidence regarding scholars’ claims on the conjoint influence of work and social contexts upon employee creativity. The investigation of the complex sets of individual–environment interactions still has theoretical significance for the study of employee creativity (Zhou & Hoever, 2014). Surprisingly, our findings suggest that conscientiousness has a positive main effect on the employee creativity of frontline technical workers and that there exists a three-way interaction among conscientiousness, perceived time pressure, and low levels of trust in one’s leader. From the interactionist perspective of organizational creativity (Zhou & Hoever, 2014), these unexpected findings illustrate that in certain contexts, the interaction of two negative factors could help develop high creativity (for an example, see Raja & Johns, 2010). This conforms to the position of Zhou and Hoever (2014), who noted “the combination of high levels of two or more positive or negative factors does not reliably yield the highest or lowest levels of creativity. In some cases, individually positive or negative actor factors cancel each other out in their effects or jointly create an effect of the reverse direction” (p. 351). Likewise, Tromp and Sternberg (2022) propose a Person × Task × Situation synergistic paradigm of dynamic creativity, suggesting variation in the extent to which each element influences the others and a degree of overlap among elements, and that the dynamic aspect of creativity is relevant to changes in different personal characteristics, for different tasks, and for different situations. In our study, it is possible that either the perceived time pressure or low levels of trust in one’s leader cannot independently stimulate or enhance employee creativity because conscientious technical employees have higher levels of creative self-efficacy to generate creative ideas (Liu et al., 2016). However, when the two contextual factors are combined together, they offer conscientious technical employees more job control to manage their work and ultimately promote creative outputs. We expect future research to continuously advance our knowledge of the intricately interactive relationship between individual characteristics and work environments associated with employee creativity by precisely capturing the nature of individual characteristics, that of work and social contexts, and that of their interactions in specific broader contexts such as professions, industries, and cultures.

## 6. Managerial Implications

This study has provided business practitioners with some interesting implications. Our findings indicate that conscientious frontline technical workers could contribute to the creative outputs of their organizations. Therefore, manufacturing enterprises need to reconsider more effective approaches to appreciate those who can work creatively. Since manufacturing enterprises may not always employ individuals with traditional creativity-relevant personality traits like openness to experience, conscientiousness might be considered as an additional human characteristic for employee creativity. Managers could provide conscientious technical employees with suitable roles and settings. For example, managers could authorize those employees to lead a team with tasks. Likewise, though conscientious employees may like to engage in challenging tasks, managers should place an appropriate workload on and give reasonably challenging requests to these conscientious technical workers when considering their work responsibility and education levels. Some technical improvements that require experience in daily work tasks are suitable requests for these conscientious employees.

To build up more supportive social contexts for these employees, organizations have traditionally developed internal training programs for their managers and helped them learn to win the trust of subordinates. However, our findings suggest that supervisors can not always expect to cultivate high levels of trust among their technical workers. On some occasions, perceptions of relatively low trust may not always be detrimental to the creative performance of those conscientious technical workers. However, this finding does not mean that managers do not build good relationships with conscientious workers. Rather, it is advisable for managers to keep a relative distance from their subordinates and enable those conscientious employees to try their best when generating creative ideas.

## 7. Limitations and Future Research

Our study has some limitations that could be considered in future studies. We only included one work characteristic to investigate how time pressure influences the consciousness–creativity relationship. Future research could take more work characteristics into consideration (e.g., job autonomy, job control, and job complexity).

A second concern relates to the generalizability of our data. Our sample predominately comprised female employees with a high school education. Future studies could extend our findings with more highly educated and gender-balanced samples. Since frontline technical workers are more likely to undertake routine tasks than creative tasks in their daily work, the creative performance may not be obvious. In addition, the structured and routine nature of the work in our sample may influence whether or not conscientiousness, instead of openness to experience, serves an important personal trait to promote employee creativity. We expect future research to examine the relationship between conscientiousness and employee creativity in other occupations such as R&D technicians and advertisement designers.

A third limitation is associated with our samples. Though our multilevel analyses extensively corrected for the cognitive biases of the supervisors’ ratings from three different manufacturing firms, we could not eliminate this influence completely. Future research could benefit from a more diverse sample to validate our findings. For instance, we collected data from three manufacturing organizations where different patterns of work designs could significantly influence the level of perceived time pressure and trust. Though we controlled for intrinsic motivation to reduce the potential impact of job characteristics on employee creativity, this may somewhat explain the reason why there is no independently significant moderating effect on the perceived time pressure and trust in one’s leader. We encourage future researchers to distribute surveys to participants who work in similar modes of job design. A broader range of occupations in future studies could examine whether the observed patterns persist across different work environments.

Also, our study provides two research avenues. First, researchers could generate fruitful findings if they continue to explore the relationship between conscientiousness and employee creativity since research to date is far from conclusive about how individual traits affect employee creativity (Anderson et al., 2014). Our study identified a positive effect of conscientiousness and employee creativity and a three-way interaction between conscientiousness, perceived time pressure, and trust in one’s leader on employee creativity. It is important to consider and reexamine these nonsignificant main effects of perceived time pressure and trust in one’s leader. In addition to a more careful examination on the two contextual factors, psychologists have noticed two dimensions of conscientiousness: achievement and dependability (Costa et al., 1991). While the former reflects an individual’s “self-centered orientation” for competence and success, the latter captures an individual’s “other-centered orientation” for responsibilities and duty (Haller & Courvoisier, 2010). Though we attempted to take both dimensions into account, we did not specify and differentiate these two dimensions in our analysis. We expect future research to look for the effect of these two facets of conscientiousness.

Second, it would be intriguing to explore cultural factors as boundary conditions that may facilitate or impede employee creativity. Existing theories and empirical evidence to explain employee creativity in different cultures are still in their inception (Anderson et al., 2014). Our study investigated how individual traits, work characteristics, and social contexts jointly influenced employee creativity in China, where individuals exhibit distinct value orientations from their Western counterparts. Could we replicate our study and find similar results in Western organizations? For example, it might be interesting to test some cultural factors (e.g., conformity and universalism) (Schwartz, 1992) to uncover whether such cultural factors moderate the interactive effect between conscientiousness, time pressure, and social contexts (e.g., trust in one’s leaders) on employee creativity. The two dimensions of conscientiousness (self- versus other-centered orientation) may have different effects on employee creativity in countries with predominantly individualistic cultures versus collectivist cultures. Given the increasing levels of cross-cultural creative collaboration, this research line would substantially deepen our knowledge of cultural contexts under which individual traits affect employee creativity.

## Figures and Tables

**Table 1:** Means, standard deviations (SDs), reliability coefficients, and correlations.

	Mean	SD	1	2	3	4	5	6	7	8	9	10	11
1. Employee creativity	4.24	1.40	0.97										
2. Conscientiousness	4.74	0.83	0.49 **	0.85									
3. Time pressure	3.61	1.27	−0.01	0.08	0.85								
4. Trust in leader	4.55	0.88	0.16 *	0.38 **	0.12 *	0.72							
5. Organization 1	0.24	0.43	0.05	0.23 **	−0.24 **	0.20 **	-						
6. Organization 2	0.35	0.48	0.44 **	0.04	−0.03	−0.25 **	−0.42 **	-					
7. Gender	0.28	0.45	−0.01	0.13 *	−0.05	0.12	0.55 **	−0.30 **	-				
8. Age	36.37	8.02	−0.18 **	0.05	−0.02	0.06	0.21 **	−0.25 **	0.00	-			
9. Organizational tenure	3.18	3.09	0.23 **	0.10	0.03	0.04	0.22 **	0.16 *	0.16 *	0.28 **	-		
10. Openness to experience	4.10	1.05	0.07	0.41 **	0.18 **	0.36 **	0.15 *	−0.35 **	0.23 **	0.05	−0.10	0.83	
11. Intrinsic motivation	4.40	1.09	0.29 **	0.53 **	0.09	0.47 **	0.15 *	−0.06	0.13 *	0.06	0.06	0.50 **	0.87

Note. N = 260. Reliabilities are in italics on the diagonal. * *p* < 0.05; ** *p* < 0.01.

**Table 2:** Results of hierarchical linear modeling analyses with employee creativity.

	Model 0	Model 1	Model 2	Model 3	Model 4	Model 5	Model 6	Model 7	Model 8
Intercept	3.85 ** (0.44)	3.85 ** (0.44)	3.85 ** (0.44)	3.88 ** (0.45)	3.88 ** (0.45)	3.85 ** (0.44)	3.86 ** (0.45)	3.86 ** (0.45)	3.86** (0.45)
Organization 1	0.56 (0.45)	0.57 (0.45)	0.57 (0.45)	0.58 (0.44)	0.59 (0.44)	0.56 (0.45)	0.56 (0.45)	0.56 (0.45)	0.59 (0.44)
Organization 2	1.23 ** (0.44)	1.22 ** (0.44)	1.22 ** (0.44)	1.25 ** (0.44)	1.25 ** (0.44)	1.22 ** (0.44)	1.23 ** (0.44)	1.24 ** (0.44)	1.29 ** (0.44)
Gender	−0.08 (0.18)	−0.08 (0.18)	−0.08 (0.18)	−0.08 (0.18)	−0.09 (0.18)	−0.08 (0.18)	−0.08 (0.18)	−0.08 (0.18)	−0.07 (0.18)
Age	−0.00 (0.01)	−0.01 (0.01)	−0.01 (0.01)	−0.00 (0.01)	−0.00 (0.01)	−0.01 (0.01)	−0.01 (0.01)	−0.01 (0.01)	−0.00 (0.01)
Organizational tenure	0.04 (0.03)	0.04 (0.03)	0.04 (0.03)	0.04 (0.03)	0.04 (0.03)	0.04 (0.03)	0.04 (0.03)	0.04 (0.03)	0.04 (0.03)
Openness to experience	−0.03 (0.05)	−0.03 (0.05)	−0.03 (0.05)	−0.03 (0.05)	−0.02 (0.05)	−0.03 (0.05)	−0.03 (0.05)	−0.03 (0.05)	−0.02 (0.05)
Intrinsic motivation	0.01 (0.04)	0.01 (0.04)	0.01 (0.04)	0.01 (0.04)	0.01 (0.04)	0.01 (0.04)	0.01 (0.04)	0.00 (0.04)	0.01 (0.04)
**Independent variables**									
Conscientiousness	0.16 * (0.07)	0.17 ** (0.06)	0.17 ** (0.06)	0.17 ** (0.06)	0.21 * (0.10)	0.15 * (0.07)	0.14 ^+^ (0.08)	0.14 * (0.07)	0.21 ^+^ (0.12)
**Moderator**									
Time pressure		−0.04 (0.04)	−0.04 (0.04)	−0.04 (0.04)	−0.03 (0.05)			−0.03 (0.03)	−0.03 (0.04)
Time pressure squared				−0.03 (0.03)	−0.02 (0.02)				−0.01 (0.02)
Trust						0.03 (0.05)	0.03 (0.05)	0.04 (0.06)	−0.03 (0.06)
**Interaction terms**									
Conscientiousness × time pressure			−0.01 (0.04)		−0.02 (0.05)			−0.02 (0.04)	−0.02 (0.04)
Conscientiousness × pressure squared					−0.03 (0.04)				−0.06 (0.05)
Conscientiousness × trust							−0.06 (0.07)	−0.08 (0.07)	0.02 (0.09)
Time pressure × trust								0.05 (0.04)	0.08 * (0.04)
Time pressure squared × trust									0.07 * (0.03)
Conscientiousness × time pressure × trust								−0.00 (0.05)	−0.02 (0.03)
Conscientiousness × time pressure squared × trust									−0.08 * (0.04)
R^2 a^	0.44	0.45	0.45	0.45	0.45	0.45	0.45	0.45	0.46
△R^2 b^		0.00	0.00	0.00	0.01	0.00	0.00	0.01	0.02

Note. Level 1 N = 260; Level 2 N = 21. Unstandardized regression weights; corresponding standard errors reported in the parentheses; substantive variable group mean-centered. ^+^ *p* < 0.1. * *p* < 0.05. ** *p* < 0.01. ^a^ Pseudo-R^2^ values represent the total within-group variance explained by the models; ^b^ △R^2^ represents the incremental variance explained over Model 0.

## Data Availability

The data presented in this study are available on request from the corresponding author.
